# Free Radical Scavenging Activity and Anthocyanin Profile of Cabernet Sauvignon Wines from the Balkan Region

**DOI:** 10.3390/molecules15064213

**Published:** 2010-06-10

**Authors:** Blaga Radovanović, Aleksandra Radovanović

**Affiliations:** 1 Department of Chemistry, Faculty of Science and Mathematics, University of Niš, Višegradska 33, P.O.Box 224, 18000 Niš, Serbia; 2 Faculty of Chemistry, Studentski trg 12-16, 1100 Belgrade, Serbia, E-Mail: radovanovic911@yahoo.com (A.R.)

**Keywords:** Cabernet Sauvignon wines, anthocyanins, free radical scavenging activity, HPLC

## Abstract

The present study is focused on anthocyanin derivatives characterizing the antioxidant activity of Cabernet Sauvignon wines produced from different vineyard regions in the Balkans. These bioactive compounds were quantified with a high performance liquid chromatography (HPLC)-diode array detection (DAD) method. The antiradical activity was estimated by the ability of the wine to scavenge the stable 2,2`-diphenyl-1-picrylhydrazyl free radical (DPPH^•^). The results show that the total anthocyanin content varied from 205.88 to 1940.28 mg/L, depending on agroclimatic factors and the enological practices of the corresponding vineyard region. The most prominent antocyanin in all investigated Cabernet Sauvignon wines was malvidin-3-*O*-monoglucoside, which accounted for 50.57% of total content, followed by its acetyl derivatives, 15.45%, and *p*-coumaryl derivatives 5.66%. The relationship between the anthocyanin derivatives and free radical scavenging activity is discussed. A high correlation between total anthocyanin content and DPPH· scavenging ability of tested wines was confirmed (r^2 ^= 0.9619). The significant correlations were obtained between antiradical activity and the sum of 3-monoglucoside (r^2 ^= 0.95594), the sum of 3-acetyl-3-glucoside (r^2 ^= 0.9728) and the sum of *p*-coumaryl-3-glucoside (r^2 ^= 0.8873) of wine samples. It can be concluded that, the anthocyanin composition can be used as biochemical marker for the authenticity of red grape cultivar and their corresponding single-cultivar wine.

## 1. Introduction

Anthocyanins, extracted from the skins of grapes during crushing, pressing, and fermentation are major components responsible for red wine color. The concentration and composition of anthocyanins in red wine grapes vary with species, variety, season, and a wide range of environmental and management factors such as climate, soil conditions and weather. Significant differences in anthocyanin concentration between sun exposed and shaded clusters of Cabernet Sauvignon grape were found by Crippen and Morrison [1]. 

On the other hand, winemaking conditions play an important role in the extraction of anthocyanins from grape and in their further stability in wine: the time of maceration and fermentation in contact with the grape skins and seeds, pressing, maturation, fining and aging conditions [2,29].

Wine consists of different phenolic compounds, so the antioxidant and the biological activity of wine are connected with a synergy of these compounds. Recent studies indicate that consumption of the small amounts of red wine on a regular base reduce the risk of coronary heart disease, and atherosclerosis, and this benefit is ascribed to the antioxidant properties of the polyphenolic compounds [3,4]. Still, it is very important to determine which group of phenolic compounds is most influential in these properties of wine. In both, *in vitro* and *in vivo* research trials, anthocyanins have demonstrated noticeable ability to reduce cancer cell proliferation and to inhibit tumor formation [5,6,7]. Finally, the higher is the concentration of phenolic compounds in wines, the higher will be theirs antioxidant capacity and antimicrobial activity [8,9,10,11]. In recent years, evidence has accumulated suggesting that wine may be one of the most prominent elements that contribute to the beneficial health effect of the so-called “the Mediterranean diet” [12]. 

Although various classes of polyphenols have been thoroughly studied with respect to their impact on quality of wines, only relatively recently have anthocyanin components gained increasing interest as nutritional antioxidants. This suggests that the anthocyanin profile, defined as the percentage content of each anthocyanin, is less strongly modified by environmental factors, such as soil irrigation, exposure to sun, degree of ripening of the grape, and can be used for verifying the varietal authenticity of grapes and their corresponding single-cultivar wines. The use of statistical models created on the basis of percentage values of singular anthocyanins has given good results in the chemotaxonomic classification of grapes of different varieties and from various geographical areas [13]. Anthocyanins are accumulated in cell vacuoles and are responsible for diverse pigmentation from orange to red, purple and blue in flowers, fruits and vegetables. Due to presence of eight conjugated double bonds carrying a positive charge, anthocyanins are intensely red or orange under acidic conditions (below pH 2), but in higher pH they are colorless and in alkaline conditions change their colour into bluish. Intensity and type of the color of anthocyanins is affected by the number of hydroxyl and methoxyl groups: if more hydroxyl groups, then the color goes toward a more bluish shade; if more methoxyl groups, then redness is increased [14]. 

Cabernet Sauvignon is the world`s most widely recognized red wine grape variety. From France, the grape spread across Europe, New World and to South Africa. Despite its importance in the world of wine, the grape is a relatively new variety, being the product of a chance crossing between Cabernet Franc and Sauvignon Blanc sometime during the 17^th^ century in southwestern France. 

The Balkan region has some good modern vineyards, 80 per cent of which are planted with red wine grapes, including Cabernet Sauvignon and Merlot, as well as the indigenous grapes. Considering the economic importance of winemaking in the Balkan countries, the aim of this work was to evaluate the free radical scavenging activity of eighth Cabernet Sauvignon wines produced from different agronomical and winemaking regions, and their correlation with the total and the real content of the main anthocyanin derivatives. 

## 2. Results and Discussion

### 2.1. The content and the distribution of anthocyanins in Cabernet Sauvignon wine samples

Anthocyanin composition is an important quality parameter for red grapes because of the significance of these compounds in determining color of the resulting wines [14]. All investigated Cabernet Sauvignon wines produced from different vineyards in the Balkan region are listed in Table 1.

**Table 1:** The wine samples studied.

Wine code	Wine and vintage	Wine producer
I	Cabernet Sauvignon- *Mezzek*, 2007	Katarzyka Estate (Bulgaria)
II	Cabernet Saugnon, 2007	Laguna (Poreč, Croatia)
III	Cabernet Sauvignon, 2007	Plantaža (Montenegro)
IV	Cabernet Sauvignon - *Alexandria*, 2007	Tikveš (Macedonia)
V	Cabernet Sauvignon - *Oplenac*, 2008	Cellars of King, (Oplenac, Serbia)
VI	Cabernet Sauvignon - *Terra Lazarica*, 2007	Rubin (Kruševac, Serbia)
VII	Cabernet Sauvignon, 2008	Radmilovac (Belgrade, Serbia)
VIII	Cabernet Sauvignon, 2008	Cevin (Niš, Serbia)

The red wines from the Balkan region were found to contain significantly higher amounts of anthocyanins [10,12,24]. The changes in content of anthocyanin composition and radical scavenging activity in investigated Cabernet Sauvignon wine samples are presented in Table 2. The total content of anthocyanins varied from 205.88 mg/L (Cabernet Sauvignon - *Terra Lazarica,* No. VI, Table 1.) to 940.28 mg/L (Cabernet Sauvignon- *Oplenac,* No. V, Table 1.), the average value being 1073.08 mg/L. High concentration of anthocyanins in Cabernet Sauvignon grape is essential for good color in Cabernet Sauvignon wine. There are numerous viticulture factors that affect grape anthocyanin content [17,18,19,20]. Light exposure to grape clusters and berry size are two of the most important [1]. The results obtained confirm a variation in the anthocyanin composition of wine samples tested. The range of the data obtained is in agreement with the available literature [15,16,17,18,19,20].

Monomeric anthocyanins are the most labile phenolic compounds in wine, typically decreasing at a rate of about 50% per year [14,25]. Anthocyanin extraction and stability are affected by winery production practices. Monomeric anthocyanins usually decline during fermentation and maceration, but the process may continue throughout the life of a wine. Wine characteristics such as pH, SO_2_, and acetaldehyde influence these processes and anthocyanin interactions with other phenolic compounds. Stability of anthocyanins can be enhanced through so called copigmentation. Copigmentation plays a crucial role in wine ageing and maturation. Acylated anthocyanins containing two or more aromatic acyl groups may affect the color through a mechanism called intramolecular copigmentation [19,20]. 

Anthocyanins also interact with other flavonoids and related compounds to produce an increase in color intensity and a shift in the wavelength of maximum absorbance toward higher wavelengths. Such a phenomenon is called intermolecular copigmentation, which can take place in acidic, neutral and even slightly alkaline aqueous solution. Polymeric anthocyanin content of the Cabernet sauvignon wine samples from the 2007 and 2008 vintage seasons (Table 2), using the pH-differential method [21] was determined from 45.50 to 69.77%. As wines age, a greater proportion of their anthocyanin content is polymerized, then color density varied from 6.76 to 11.56 (mean 7.16) and hue from 0.62 to 1.12 (mean 0.96) absorbance units. 

**Table 2:** Some analytical data, content of anthocyanins and radical scavenging activity of the wine samples.

Wine code	Alcohol (vol %)	pH value	Total anthocyanins (mg/L)	Polymeric color (%)	Color density (absorbance units)	Hue (absorbance units)	Radical scavenging (%)
I	14.0	3.67	593.36 ± 2.14	69.08 ± 0.15	8.44 ± 0.25	1.11 ± 1.20	75.05 ± 1.20
II	12.0	3.55	424.66 ± 1.45	63.45 ± 0.23	7.86 ± 1.02	0.93 ± 0.45	71.30 ± 0.45
III	12.5	3.45	505.17 ± 2.55	65.86 ± 0.22	8.23 ± 1.06	1.12 ± 0.23	72.06 ± 0.23
IV	11.5	3.21	408.26 ± 2.09	54.88 ± 0.22	6.76 ± 0.25	1.06 ± 0.16	71.10 ± 0.16
V	13.0	3.41	1940.28 ± 2.33	45.50 ± 0.16	11.56 ± 0.34	1.03 ± 0.56	83.53 ± 0.56
VI	11.5	3.54	205.58 ± 1.10	69.77 ± 0.23	6.88 ± 0.28	0.82 ± 1.02	70.03 ± 1.02
VII	12.5	3.54	850.08 ± 1.45	66.76 ± 0.17	8.78 ± 0.15	1.01 ± 1.12	76.16 ± 1.12
VIII	11.5	3.85	335.44 ± 2.05	68.15 ± 0.13	7.21 ± 0.24	0.94 ± 0.75	70.87 ± 0.75

### 2.2. HPLC analysis of Cabernet Sauvignon wine samples

Anthocyanins profile of grape and wine, determined by the relative proportions of the different anthocyanins, are characteristic for each grape variety and wine, corresponding. Moreover, concentrations of different compounds can vary significantly within grape cultivars according to environmental conditions and winemaking practices [1,20,29]. 

**Figure 1:**
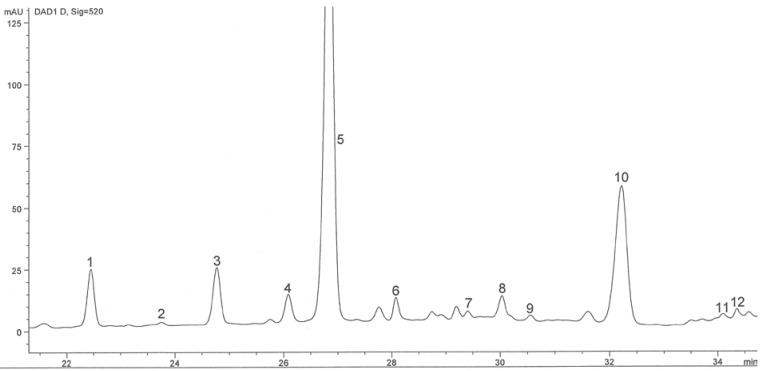
HPLC chromatogram of anthocyanins in Cabernet Sauvignon wine from Oplenac viticulture region (Serbia) monitored at 520 nm.

Anthocyanins were identified by their retention times, which were compared to standards, since that is their characteristic wavelength. The chromatographic of Cabernet Sauvignon wine sample, Oplenac vineyard region in Serbia monitored at 520 nm is given in Figure 1. In this chromatogram the peaks 1, 2, 3, 4 and 5 were identified as -3-glucosides: delphinidin- (1), cyanidin- (2), petunidin- (3), peonidin- (4) and malvidin-3-glucoside (5); the peaks 6, 7, 8, 9 and 10 were identified as -3-glucoside-acetates: the delphinidin- (6), cyanidin- (7), petunidin- (8), peonidin- (9) and malvidin-3-glucoside acetate (10), and the peaks 11, 12 and 13 were identified as -3-glucoside-*p*-coumarates: petunidin- (11), peonidin- (12) and malvidin-3-glucoside-*p*-coumarate (13):

The quantification of results for anthocyanin derivatives in the analyzed samples is shown in Table 3. In *Vitis vinifera* L. red cultivars there are only delphinidin (Dp), cyanidin (Cy), petunidin (Pt), peonidin (Pn) and malvidin (Mv)-3-glucosides, along with the corresponding acetyl, p-coumaryl derivatives. Cyanidin is the precursor pigment of the other anthocyanidins, and it can be transformed into peonidin by the action of a 3`-O-methyltransferase, or into delphinidin by the action of a 3`-hydroxylase. A 3`-5`-O-methyltransferase transforms delphinidin into petunidin, and petunidin into malvidin [19]. 

**Table 3:** Content of individual anthocyanins and total anthocyanin content of the wines samples (mg/L ± SD, *n* = 3).

Antocy.	W. code							
	I	II	III	IV	V	VI	VII	VIII
Dp-3-gl (1)	21.50 ± 0.17	24.67 ± 0.21	18.48 ± 0.67	20.20 ± 0.87	83.86 ± 0.45	10.63 ± 1.09	37.39 ± 1.54	7.02 ± 1.09
Cy-3-gl (2)	n.d.	n.d.	6.75 ± 0.99	9.26 ± 1.11	6.17 ± 0.98	7.33 ± 1.12	n.d.	n.d.
Pt-3-gl (3)	27.61 ± 1.02	32.80 ± 0.95	34.48 ± 1.76	29.85 ± 0.97	87.83 ± 0.45	14.48 ± 0.20	41.55 ± 0.91	12.97 ± 65
Pn-3-gl (4)	22.44 ± 0.05	10.81 ± 0.86	35.87 ± 2.00	30.47 ± 0.34	44.25 ± 0.67	10.59 ± 0.44	30.19 ± 1.23	5.43 ± 0.43
Mv-3-gl (5)	300.62 ± 0.01	248.24 ± 1.10	249.19 ± 1.97	187.11 ± 1.09	883.13 ± 0.99	103.04 ± 0.56	461.90 ± 1.05	168.52 ± 0.97
Dp-3-gl- ac (6)	18.15 ± 1.11	12.14 ± 1.02	16.96 ± 1.56	24.32 ± 1.12	28.52 ± 0.78	4.24 ± 0.95	15.00 ± 1.01	36.86 ± 0.45
Cy-3-gl- ac (7)	8.97 ± 0.55	6.60 ± 1.45	6.64 ± 0.67	9.94 ± 0.31	15.15 ± 0.55	5.95 ± 0.97	9.35 ± 0.97	23.09 ± 1.14
Pt-3-gl -ac (8)	8.83 ± 0.65	9.10 ± 0.65	8.57 ± 0.97	9.40 ± 0.75	45.48 ± 1.04	6.86 ± 0.35	16.25 ± 0.23	8.30 ± 1.25
Pn-3-gl- ac (9)	7.28 ± 0.75	5.50 ± 0.97	11.27 ± 1.43	8.02 ± 0.98	10.03 ± 1.10	3.10 ± 0.13	11.10 ± 0.75	n.d.
Mv-3-gl-ac (10)	96.87 ± 0.23	56.04 ± 1.15	46.20 ± 2.03	49.45 ± 1.09	319.03 ± 1.09	32.43 ± 0.99	149.75 ± 0.66	87.17 ± 0.95
Pt-3-gl- p-co ( 11)	18.63 ± 0.43	n.d.	17.42 ± 1.23	11.23 ± 1.23	31.38 ± 0.99	7.81 ± 1.08	10.53 ± 0.56	n.d.
Pn-3-gl-p-co (12)	9.53 ± 0.02	5.47 ± 1.23	15.26 ± 1.95	12.81 ± 1.43	44.03 ± 0.87	n.d.	11.77 ± 0.78	n.d.
Mv-3-gl- p-co (13)	40.07 ± 0.05	18.77 ± 0.67	38.18 ± 0.67	22.59 ± 0.56	138.53 ± 0.98	13.29 ± 1.09	39.06 ± 1.07	9.53 ± 0.89
Sum-3-gl	372.18 ± 0.12	316.52 ± 1.85	344.78 ± 1.35	276.89 ± 0.54	1175.03 ± 3.12	146.07 ± 1.21	570.03 ± 0.98	186.92 ± 1.65
Sum-3-gl-ac	140.10 ± 2.05	89.38 ± 2.57	89.64 ± 3.10	101.13 ± 1.97	418.21 ± 2.14	52.58 ± 1.13	201.45 ± 1.98	68.34 ± 2.05
Sum-3-gl-p-co	68.23 ± 3.09	24.24 ± 3.10	70.86 ± 2.15	46.63 ± 2.76	213.94 ± 2.00	21.10 ± 2.12	61.36 ± 2.00	9.53 ± 1.15

Dp-, Cy-, Pt-, Pn-, Mv-: delphinidin-, cyanidin-, petunidin-, peonidin- and malvidin-3- monoglucoside; -ac: -acetate; p-coum: -p-coumarate, respectively. Sum-3-gl: sum of non-acylated anthocyanins; sum-3-gl-ac: sum of acetates and sum-3-gl-co: sum of p-coumarates. The symbol n.d.: not detected.

Quantitatively, the content of glucosylated antocyanins was predominant in the investigated Cabernet Sauvignon wines (mean amount of content was 423.55 mg/L – from 146.07 to 1175.03 mg/L), followed by the content of acetylated ones (mean amount was 145.10 mg/L – from 52.58 to 418.21 mg/L) and the content of *p*-coumarylated ones (mean amount was 64.49 mg/L – from 9.53 to 213.94 mg/L). The contribution of each anthocyanin to the final anthocyanin profile was calculated based on the monoglucoside forms and is expressed as percentage in Table 4.

**Table 4:** Distribution of individual anthocyanins and total anthocyanin contents of the wines samples (% ± SD, *n* = 3).

Anthoc.	W. code							
	I	II	III	IV	V	VI	VII	VIII
Dp-3-gl (1)	3.62 ± 0.21	5.81 ± 0.45	3.66 ± 0.23	4.96 ± 0.55	4.32 ± 0.12	5.17 ± 0.11	4.40 ± 0.23	2.09 ± 0.13
Cy-3-gl (2)	n.d.	n.d.	1.33 ± 0.07	2.27 ± 0.09	0.32 ± 0.02	3.57 ± 0.09	n.d.	n.d.
Pt-3-gl (3)	4.65 ± 0.01	7.72 ± 1.02	6.83 ± 0.06	7.33 ± 0.10	4.53 ± 0.06	7.04 ± 0.04	4.89 ± 0.96	3.87 ± 0.06
Pn-3-gl (4)	3.78 ± 0.04	2.55 ± 0.90	7.10 ± 0.15	7.48 ± 0.15	2.28 ± 0.18	5.15 ± 0.55	3.55 ± 0.85	1.62 ± 0.01
Mv-3-gl (5)	50.66 ± 0.10	58.46 ± 0.21	49.33 ± 0.16	45.93 ± 0.55	45.36 ± 0.15	50.12 ± 0.56	54.43 ± 0.06	50.24 ± 1.09
Dp-3-gl (6)	3.06 ± 0.05	2.86 ± 0.55	3.36 ± 0.45	5.96 ± 0.23	1.47 ± 0.92	2.06 ± 0.09	1.76 ± 0.05	10.99 ± 0.98
Cy-3-gl-ac (7)	1.51 ± 0.03	1.55 ± 0.43	1,31 ± 1.02	2.43 ± 0.12	0.78 ± 1.00	2.89 ± 0.05	1.10 ± 0.02	6.88 ± 1.02
Pt-3-gl-ac (8)	1.49 ± 0.10	2.14 ± 1.02	1.70 ± 0.91	2.30 ± 0.16	2.34 ± 0.96	3.34 ± 0.06	1.91 ± 0.01	2.47 ± 0.14
Pn-3-gl-ac (9)	1.23 ± 0.40	1.30 ± 1.00	2.23 ± 0.23	1.96 ± 0.05	0.52 ± 0.55	1.51 ± 0.04	1.31 ± 0.02	n.d.
Mv-3-gl-ac (10)	16.33 ± 0.12	13.20 ± 1.09	9.15 ± 0.07	12.11 ± 0.17	16.44 ± 0.17	15.77 ± 0.45	17.62 ± 0.05	25.99 ± 0.06
Pt-3-gl-p-co (11)	3.14 ± 0.05	n.d.	3.45 ± 0.00	2.75 ± 0.03	1.62 ± 0.11	3.80 ± 0.58	1.24 ± 0.55	n.d
Pn-3-gl-p-co (12)	1.61 ± 0.07	1.29 ± 0.08	3.02 ± 0.05	3.14 ± 0.01	2.27 ± 0.08	n.d.	1.38 ± 0.23	n.d.
Mv-3-gl-p-co (13)	6.75 ± 0.06	4.42 ± 0.06	7.56 ± 0.17	5.53 ± 0.05	7.14 ± 0.05	6.46 ± 1.02	4.59 ± 0.12	2.84 ± 0.89
Sum-3-gl	63.22 ± 0.15	74.53 ± 0.09	68.25 ± 0.45	67.82 ± 0.67	60.56 ± 0.56	71.05 ± 0.55	67.06 ± 0.78	55.72 ± 0.09
Sum-3-gl -ac	23.61 ± 0.12	21.05 ± 0.11	17.74 ± 1.01	24.77 ± 0.96	21.55 ± 0.06	25.58 ± 0.95	23.70 ± 0.98	20.37 ± 0.21
Sum-3-gl-p-co	11.50 ± 0.22	5.71 ± 0.10	14.03 ± 1.05	11.42 ± 1.09	11.03 ± 0.66	10.26 ± 1.00	7.22 ± 1.09	2.84 ± 0.23

Dp-, Cy-, Pt-, Pn-, Mv-: delphinidin-, cyanidin-, petunidin-, peonidin- and malvidin-3- monoglucoside; -ac: -acetate; p-coum: -p-coumarate, respectively. Sum-3-gl: sum of non-acylated anthocyanins; sum-3-gl-ac: sum of acetates and sum-3-gl-co: sum of p-coumarates. The symbol n.d.: not detected.

The distribution of the most common anthocyanins in the investigated Cabernet Sauvignon wines also is depending of the microclimatic factors of region and winemaking technique: Delphinidin- was determined from 2.09 to 5.81% (mean 3.95%); Cyanidin- varied from 0.32 to 3.57% (mean 1.94%); Petunidin- was determined from 3.38 to 7.33% (mean 5.85%) and peonidin-3-O-glucoside varied from 1.62 to 7.48% (mean 4.55%). The most prominent anthocyanin was malvidin-3-O-monoglucoside, which accounted for 50.57% (from 45.36 to 58.46%) of total content, followed by its acetyl derivatives, 15.45% (from 9.15 to 25.99%) and *p*-coumaryl derivatives 5.66% (from 2.84 to 7.56%) in all investigated Cabernet Sauvignon wines.

The findings showed that malvidin-3-O-monoglucoside was the most abundant anthocyanin in all wines that had a prevalence of tri-hydroxylated anthocyanins. By contrast, cyanidin was the least abundant anthocyanin pigment, as demonstrated for a number of other wines [12,13,14,15,16,17,18,26]. A similar profile has been reported for Syrah [18,22], Hellenic native grape varieties [12], and Corsica red wines [13], but different patterns were observed for Cabernet Franc and Pinot Noir wines from British Columbia [3]. Another point worth mentioning is that the order of abundances based on average value of distribution for each anthocyanin was the following:

Mvgl > Mvgl-ac > Ptgl > Pngl > Dpgl > Mvgl-p-coum > Cygl

The Cabernet Sauvignon wines have a percentage of methoxylated (peonidin, petunidin and malvidin, sum 62.54%) higher than non-methoxylated (cyanidin and delphinidin, sum 6.12%) anthocyanin forms. With regard to the acylation of the glycosyl group, the distribution of non-acylated anthocyanins, mean 66.03% (from 55.72 to 74.53%) were the most abundant fraction in all wine samples. The mean amount of contribution of acetylated anthocyanins was 22.30% (from 17.74 to 25.58%), followed by coumarylated 9.25% (from 2.84 to 14.03%) in all wines. According to the wine sample results, it is obviously that the content of anthocyanin constituents of single-cultivar wines coming from different viticulture regions and wine producers were different, which may be related to the thickness of the grape skin, the climate that the grape grows in, the degree of ripeness of the grape, the application of different vinification techniques and the wine’s ages [1,2,29].

### 2.3. The correlation between the radical scavenging activity and anthocyanin derivatives of the Cabernet Sauvignon wine samples

The antioxidant activity of the wine samples was estimated by the ability of the sample to scavenge the stable DPPH• free radical [10,16,23,24]. The decrease in absorbance at 517 nm is taken as a measure of the extent of radical-scavenging. All wines show a higher DPPH• radical scavenging activity (Table 2) in the range from 70.03 to 83.53% (mean 73.76%). Anthocyanins are considered very good antioxidant agents, their high activity being attributed to their peculiar structure, namely the oxonium ion in the C ring [24]. But the number of sugar residues at the 3-position, the oxidation state of the C ring [25], the hydroxylation and methylation pattern, as well as the acylation by phenolic acids [16] are considered crucial factors for the expression of antioxidant effects. 

**Figure 2:**
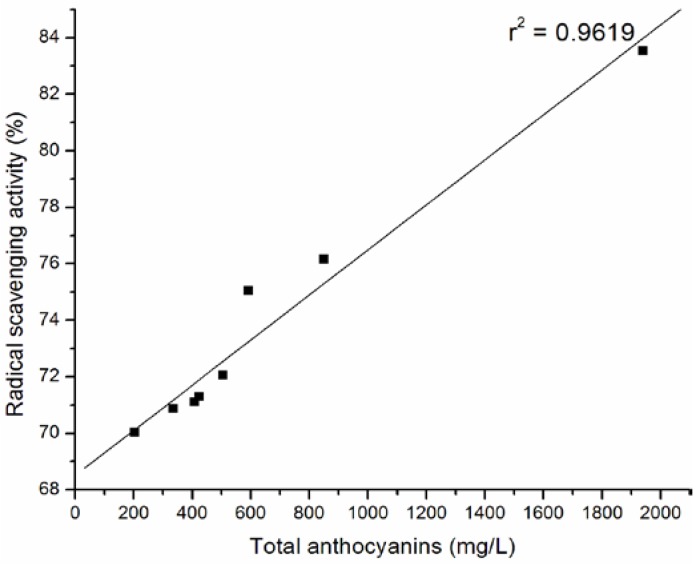
Relationship between radical scavenging activity and total anthocyanins of selected Cabernet Sauvignon wine samples.

**Figure 3:**
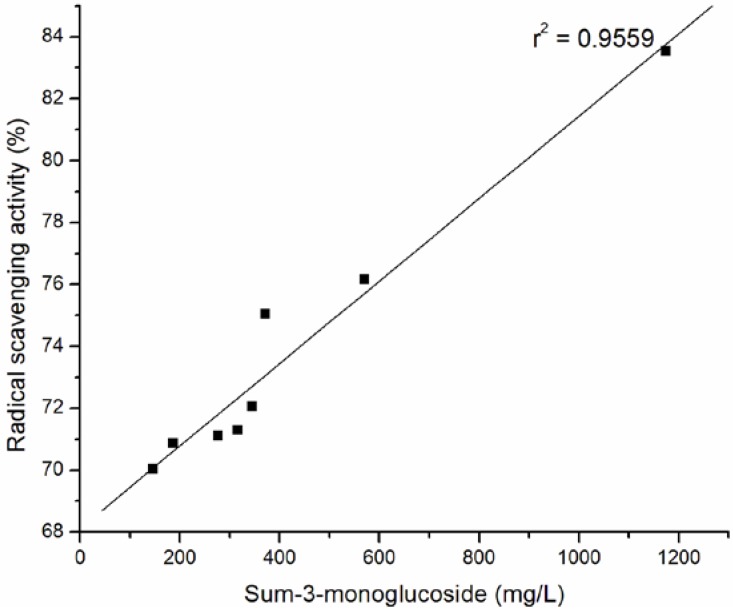
Relationship between radical scavenging activity and sum of non-acylated anthocyanins of selected Cabernet Sauvignon wine samples.

**Figure 4:**
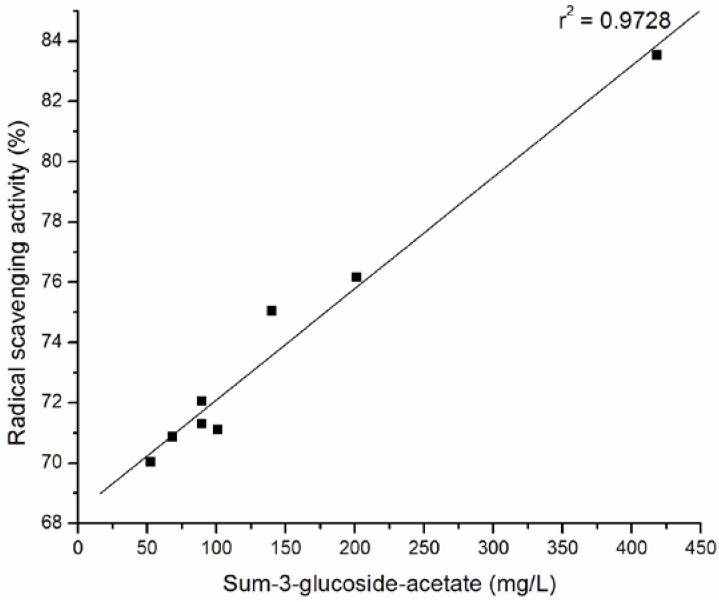
Relationship between radical scavenging activity and sum of glucoside-acetates of selected Cabernet Sauvignon wine samples.

**Figure 5:**
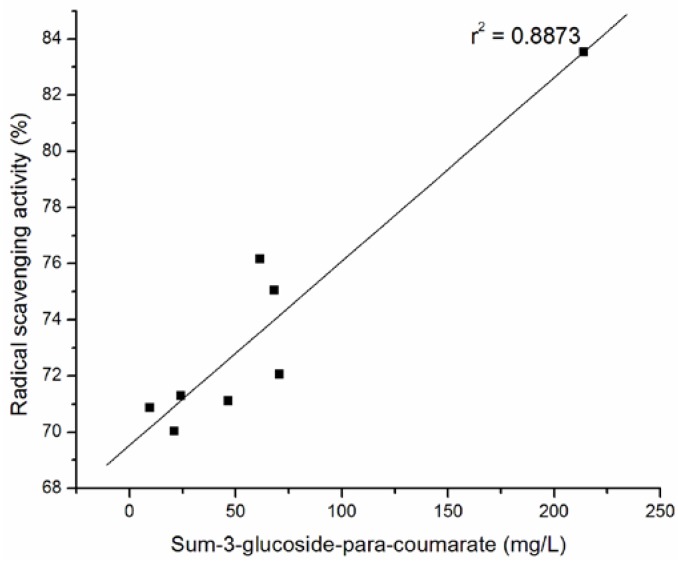
Relationship between radical scavenging activity and sum of glucoside-*p*-coumarates of selected Cabernet Sauvignon wine samples

The percentage of DPPH radical scavenging activity against the content of the total anthocyanins, the sum of 3-monoglucoside, the sum of 3- acetyl-3-glucoside and the sum of *p*-coumaryl-3-glucoside of wine samples are plotted in Figure 2 , Figure 3, Figure 4, and Figure 5.

The strong correlation between total anthocyanins and DPPH· scavenging ability (r^2 ^= 0.9619) of tested wines was confirmed. The significant correlations were obtained between antiradical activity and the sum of 3-monoglucoside (r^2 ^= 0.95594), the sum of 3- acetyl-3-glucoside (r^2 ^= 0.9728) and the sum of p-coumaryl-3-glucoside (r^2 ^= 0.8873) of wine samples. 

## 3. Experimental

### 3.1. Materials

Acetonitrile and acetic acid of HPLC-grade were obtained from Merck (Darmstadt, Germany); HPLC-grade methanol was purchased from Carlo Erba Reagent (Milan, Italy). Potassium meta-bisulfite was purchased from Merck (Darmstadt, Germany); 2,2`-diphenyl-1-picrylhydrazyl (DPPH) free radical was obtained from Sigma Chemical Co. (St. Louis, MO); Malvidin-3-monoglucoside chloride (oenin chloride ref. 0911S) from Extrasynthese (Genay, France). The used reagents were of analytical quality. 

### 3.2. Wine samples

Eight selected Cabernet Sauvignon wines with definite geographical origin, from the 2007 and 2008 vintage seasons, produced from different agronomical and winemaking regions in the Balkans, were analyzed. All of them are listed in Table 1. 

### 3.3. Determination of indices for anthocyanin pigment degradation, polymeric color, browning, and hue

Indices for anthocyanin degradation of the wine can be derived using the pH-differential method described by Giusti and Wrolstad [21]. The absorbance at 420 nm of the disulphide treated sample serves as an index for browning. The color density of the control sample and the polymer color of the disulphide bleached sample wine are calculated as follows:

Color density = [(*A*_420nm_ – *A*_700nm_) + (*Aλ*_max_ – *A*_700nm_)]

The value of hue is calculated as follows:

Hue = [(*A*_420nm_ – *A*_700nm_)/ (*Aλ*_max_ – *A*_700nm_)]

The ratio between polymeration color and color density is used to determine the percentage of the color that is contributed by polymerized material:

Polymeric color (%) = (polymeric color/color density) × 100

### 3.4. Free radical scavenging activity

The free radical scavenging activity of the wine samples was analyzed by using the 2,2′-diphenyl-1-picrylhydrazyl (DPPH^•^) assay [10,23,24] for antioxidant activity which is based on measurement of the loss of DPPH^•^ color by change of absorbance at 517 nm caused by the reaction of DPPH^• ^with tested wine sample. The reaction was monitored by a UV/VIS spectrophotometer. The diluted wine sample (wine was diluted with water, 1:10 v/v) and fresh 1x10^-4^ M DPPH^• ^metabolic solution were putted into a cuvette at the room temperature. After 20 min incubation period at room temperature, the absorbance was read against a blank at 517 nm. Scavenging capacity of DPPH^• ^in percent (%) of each wine sample was calculated from the decrease of absorbance according to the relationship:

Radical scavenging activity (%) = (1 - *A*_sample_ - *A*_blank_/*A*_control_) × 100

where *A*_control_ is the absorbance of control reaction (3x10^-4^ M DPPH• solution), *A*_blank_ is the absorbance of dilution wine sample and *A*_sample_ is the absorbance of the dilution wine sample with same concentration of DPPH• solution. Three analytical replicates were carried out on each sample wine.

### 3.5. High performance liquid chromatography (HPLC-DAD) analysis

Anthocyanins were analyzed by direct injection of the samples, previously filtered through a 0.45 µm pore size membrane filter, in an Agilent Technologies 1200 chromatographic system equipped with a Agilent photodiode array detector (DAD) 1200 with RFID tracking technology for flow cells and UV lamp, an automatic injector, and a ChemStation software. The column was termostated at 30 ^o^C. After injecting 5 µL of wine sample, separation was performed in an Agilent-Eclipse XDB C-18 4.6 × 150 mm coumn. The HPLC grade solvents used were formic acid/water as solvent A and acetonitrile/formic acid/water as solvent B. The elution profile was as follows: from 0 to 28 min, 0.0% B, from 28 to 35 min, 25% B, from 35 to 40 min, 50% B, from 40 to 45 min, 80% B, and for last 10 min again 0% B. The detection wavelength was 520 nm. The different anthocyanin compounds were identified by comparing their retention times and spectral characteristics with data given in the literature [18,20,27,28]. Quantitation was made by means of a calibration curve obtained by injecting standard solutions of malvidin-3-monoglucoside chloride with different concentrations. The range of the linear calibration curve (r^2^ was 0.9991). Results were expressed as mg/L sample.

### 3.6. Statistical analyses

Three analytical replicates were carried out on each sample wine. Measurements were averaged, and results are given as mean ± standard deviation (SD). Correlation between the anthocyanin content and antiradical efficiency was established using regression analysis at a 95% significance level (P ≤ 0.001).

## 4. Conclusions

From the results obtained, we can conclude that the amount of anthocyanins is important for understanding of antioxidant potency of red wines. The mechanism by which anthocyanins are absorbed and metabolized in the body is currently unclear. The high anthocyanin content in the investigated Cabernet Sauvignon wines contributes to its increased radical scavenging activity. It can be concluded that, the anthocyanin composition can be used as biochemical marker for the authenticity of red grape cultivar and their corresponding single-cultivar wine. 
